# A Rare Case of Duodenal Adenocarcinoma With Brain Metastasis

**DOI:** 10.7759/cureus.54202

**Published:** 2024-02-14

**Authors:** Hariharasudan Mani, Alisha Hossain, Elsie Lee, Muhammad Rizvi

**Affiliations:** 1 Hematology Oncology, Lehigh Valley Cancer Institute, Allentown, USA; 2 Hematology and Medical Oncology, Lehigh Valley Cancer Institute, Allentown, USA; 3 Pathology, Lehigh Valley Health Network, Allentown, USA; 4 Hematology and Oncology, Lehigh Valley Cancer Institute, Allentown, USA

**Keywords:** gi malignancy, chemotherapy, small bowel cancer, brain metastasis, duodenal cancer, adenocarcinoma

## Abstract

Small bowel malignancies are relatively rare, accounting for only 3% of all gastrointestinal cancers. Duodenum is the most common location among small bowel cancers, followed by Jejunum and then Ileum. Duodenal adenocarcinoma produces vague symptoms, leading to late presentation and a poor prognosis compared to similarly staged colon cancer. It is rare to have brain metastasis in duodenal adenocarcinoma, and not many case reports have been reported. Only approximately 6% of patients with gastrointestinal malignancy have brain metastasis. Here, we present a case of a 64-year-old female patient diagnosed initially with stage IV duodenal adenocarcinoma presenting with duodenal mass, abdominal lymphadenopathy, and liver metastasis. She had excellent systemic control for over two years with systemic chemotherapy, with a close to complete response on follow-up imaging. She presented with a 2 cm left frontal mass biopsy consistent with duodenal adenocarcinoma metastasis. She underwent resection of the left frontal tumor and gamma knife to the resection cavity. She continues to have good systemic control of disease. This case highlights the rare possibility of brain metastasis with duodenal adenocarcinoma, especially in patients who have good systemic control with chemotherapy.

## Introduction

Small bowel cancers of the duodenum, jejunum, and ileum are relatively rare [1]. Small bowel adenocarcinoma (SBA) is the second most common histology after carcinoid tumors in the small bowel [2]. Duodenum is the most common location among small bowel adenocarcinomas, followed by Jejunum and then Ileum [3]. Compared to colon cancer with similar stages, five-year disease-specific survival rates for SBA are worse, especially in node-positive disease [4]. Brain metastasis is rare in gastrointestinal malignancies, accounting for about 6.2% of cases [5]. Not many case reports have been reported on duodenal cancer presenting with brain metastasis. Here, we present a patient with metastatic duodenal adenocarcinoma treated with chemotherapy with good systemic control, presenting with brain metastasis.

## Case presentation

**Figure 1:**
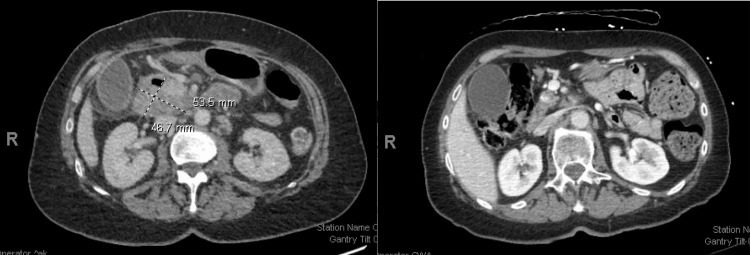
The first figure shows the initial abdominal CAT scan with 5.4cm by 4.7 cm of mass in the second and third portions of the duodenum, indicating contiguous involvement of the uncinate process. The second figure shows the follow-up CAT scan after two years with complete resolution of duodenal mass

**Figure 2:**
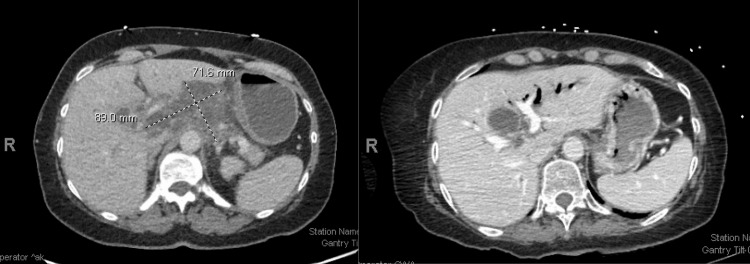
The first figure shows the initial abdominal CAT scan with an 8.9cm x 7.2cm liver metastasis within the caudate lobe. The second figure shows the CAT scan after two years with complete resolution of the liver metastasis

A 64-year-old female with a history of Stage IV metastatic duodenal adenocarcinoma to the liver (T3N2M1) presented with syncope in the setting of a likely seizure. She was initially diagnosed with duodenal adenocarcinoma after presenting with abdominal pain, and imaging revealed a duodenal mass with liver metastasis (Figures 1, 2). PET imaging demonstrated an intensely hypermetabolic mass involving the second and third portions of the duodenum, as well as extensive hypermetabolic peripancreatic, gastro-hepatic, porta hepatis, and para-aortic adenopathy, as well as subcarinal and right inferior pulmonary ligament adenopathy. Hypermetabolic liver metastasis was also seen on PET imaging. A biopsy of the duodenal mass demonstrated duodenal adenocarcinoma with normal mismatch repair (MMR) expression, HER2 negativity, and no targetable mutations on next-generation sequencing. She was initially treated with the FOLFOX regimen (Oxaliplatin 85 mg/m2, Leucovorin, Fluorouracil 400 mg/m2 bolus injection followed by 2400 mg/m2 46-hour intravenous infusion) along with Bevacizumab 5 mg/kg, repeated every two weeks, and then transitioned to 5-Fluorouracil and Bevacizumab maintenance. She has been on treatment for over two years with a complete response on follow-up imaging (Figures 1, 2).

**Figure 3:**
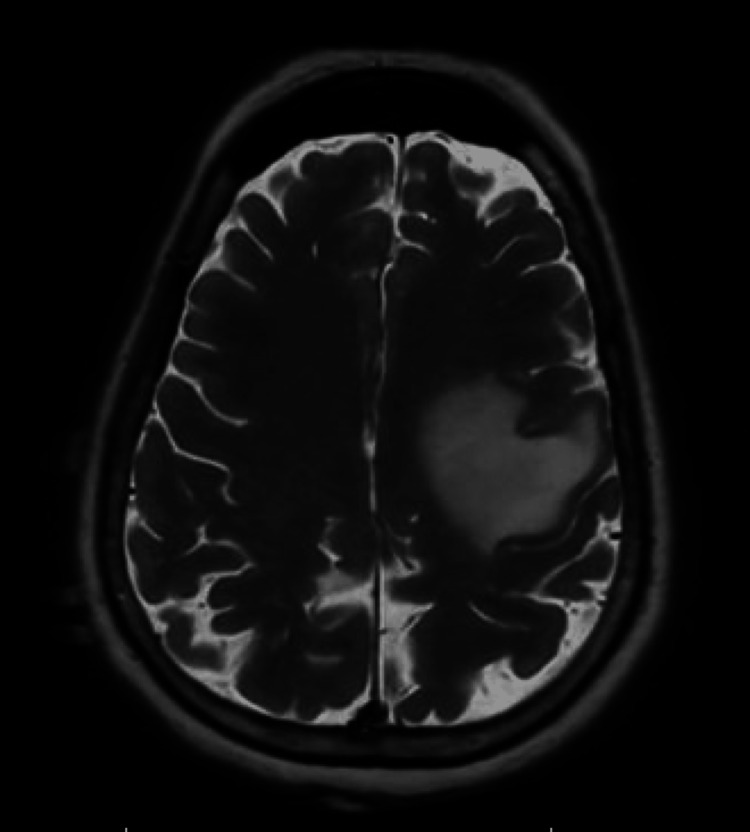
MRI brain: The T2-weighted image shows moderate to marked secondary vasogenic edema, and there is effacement of overlying cortical sulci

She presented to the emergency department after being found unresponsive and stiff by a family member at home. Thought to be a post-ictal state. The MRI brain demonstrated a new 19 x 20 mm mass at the gray-white interface of the left frontal lobe with marked vasogenic edema (Figure 3). She underwent resection of the left frontal tumor and received gamma knife treatment for the tumor cavity.

**Figure 4:**
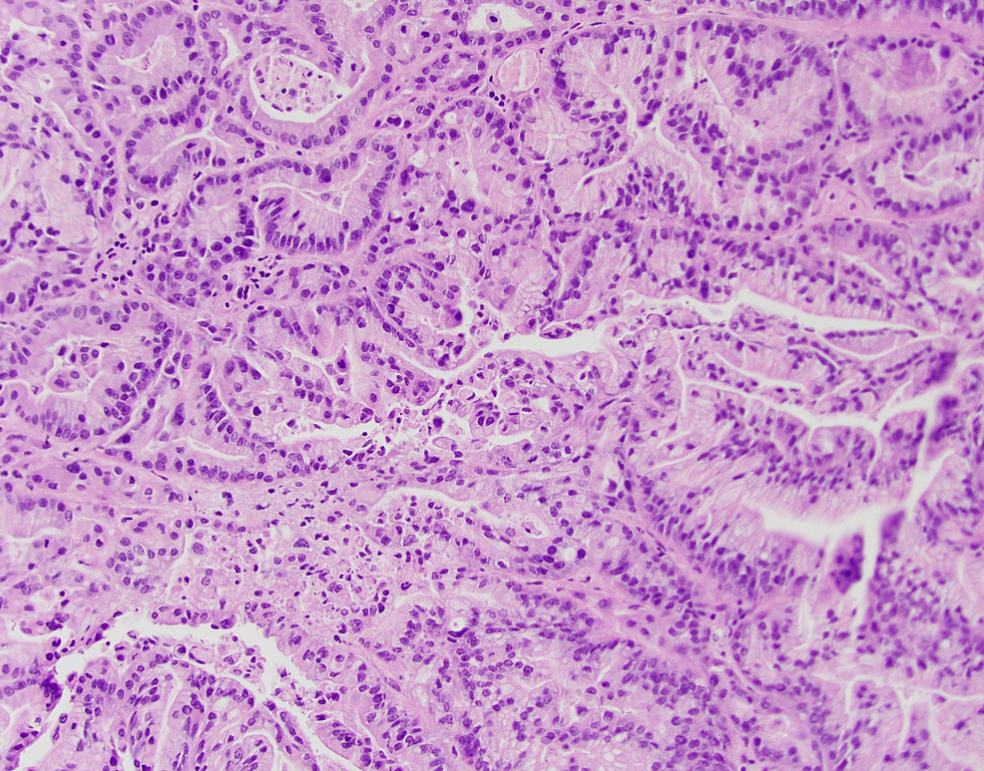
Biopsy of brain mass. On microscopic examination at high magnification, the neoplasm is composed of tall columnar epithelial cells with round to elongated nuclei arranged in complex back-to-back glandular structures with luminal necrosis. The cells show nuclear pleomorphism and pseudostratification, loss of polarity, and abundant cytoplasm.

A biopsy of the left frontal tumor was performed, and two fragments of tan-pink soft tissue, 1.0 x 0.9 x 0.8 and 1.1 x 0.6 x 0.4 cm, were submitted for intraoperative consultative diagnosis. Approximately 1/3 of the specimen was submitted for frozen section, and a diagnosis of metastatic adenocarcinoma was made. The tissue was submitted for histologic processing. On examination of the prepared H and E slides, the neoplasm showed features of a moderately differentiated adenocarcinoma compatible with an intestinal origin. On immunohistochemical stains, the tumor cells are strongly and diffusely positive for CK7, and the tumor nuclei are focally positive for CDX2 and negative for TTF1. The cells are negative for CK20. The immunoprofile is consistent with metastasis from a primary duodenal adenocarcinoma. CT of the chest, abdomen, and pelvis (Figure 4) revealed a decrease in the size of the retroperitoneal lymphadenopathy, and prior hepatic lesions were no longer measurable. She was started back on treatment with FOLFOX and Bevacizumab.

## Discussion

Small bowel cancers of the duodenum, jejunum, and ileum are relatively rare, accounting for 3% of cancers among gastrointestinal cancers [1]. Small bowel adenocarcinoma (SBA) is the second most common histology after carcinoid tumors, comprising about 30-40% of cases [2]. Duodenum is the most common location among SBAs, accounting for 52-58% of cases [3]. Opposed to the trend in other gastrointestinal malignancies, small bowel cancer incidence shows an uptrend with an annual percent increase of 1.8 between 2006 and 2015 for unclear reasons [1]. Due to the rarity of the diagnosis, treatment of metastatic SBA is based on retrospective and phase II data. Typically, metastatic small bowel adenocarcinoma is managed similarly to colon adenocarcinoma guidelines, with an emphasis on either FOLFOX, CAPEOX, FOLFIRI, or FOLFIRINOX regimens combined with bevacizumab [2]. For tumors with dMMR and high TMB (≥ 10 mutations/megabase), checkpoint inhibitors could be an option as well. (pembrolizumab [6] or Nivolumab [7] with or without Ipilimumab [8], Dostarilimab [9]. For subsequent lines of treatment, either taxane-based regimens [10], Larotrectinib or Entrectinib [11] (for neurotrophic tyrosine receptor kinase (NRTK) gene fusion), or Selpercatinib [12] (for rearranged transfection (RET) gene fusion) are options.

Among patients diagnosed with SBA, approximately 32% of patients have stage IV disease at diagnosis [13]. The most common sites of metastatic disease are the peritoneal spread and liver, similar to other GI malignancies [14]. A five-year overall survival of SBA is around 85% for localized disease and only 42% for stage IV disease [1]. Recurrence rates for localized SBA after surgery are also higher [3]. Five-year disease-specific survival rates for SBA are worse than similarly staged colon cancers, especially in node-positive disease [4]. Certain patients with limited metastasis to visceral organs could be candidates for metastatectomy and need the involvement of multidisciplinary care [15].

Gastrointestinal malignancies rarely cause brain metastasis. In a retrospective analysis, 6.2% of patients with gastrointestinal malignancies were associated with brain metastasis, the common primaries being the rectum and colon [5]. In the same study, it was also noted that patients with gastrointestinal malignancy more often present with solitary brain metastasis than patients with other primary tumors [5]. The mean duration of onset of brain metastasis in upper gastrointestinal cancers is 9.3 months in advanced disease [16]. Brain metastasis is associated with poor overall survival. Median survival after the diagnosis of brain metastasis in gastrointestinal malignancy is 3.07 months [16].

Brain metastasis from SBA is relatively rare, and from our search, we could not find any reported cases of duodenal adenocarcinoma with proven metastasis to the brain. One case report reported a brain mass and biopsy-proven duodenal primary in an 84-year-old female patient. In that report, brain mass was not biopsy-proven as the patient opted for a palliative approach due to her elderly age [17]. Another case report reported the de novo presentation of jejunal adenocarcinoma with brain metastasis [15]. The patient had a metastatectomy and a jejunal segmental resection. Further chemotherapy information was not provided. The patient died in eight months due to recurrent brain metastasis. Another case report reported brain metastasis in jejunal adenocarcinoma in a 54-year-old patient [18].

Our patient was treated with surgery followed by stereotactic brain radiation therapy in the surgical cavity. Brain metastasis is a late manifestation of GI cancer and could see an increase in incidence in the future with continuing efforts to have good systemic control and better systemic treatment options. Early detection is a better prognostic factor, as early treatment leads to better outcomes than untreated brain metastasis [16].

The cause of the rare occurrence of brain metastasis in duodenal adenocarcinoma is unclear. The exact nature of this biological behavior is unclear. One study hypothesized that Her2 expression, typically seen in adenocarcinomas, could be correlated with increased brain metastasis [19]. Brunner et al. noted that among 827 patients with gastric and esophageal cancers, HER2 status was positive (2+ or 3+) in 18.5% and negative in 37.03% of patients with brain metastasis. 44.44% of patients had missing data [16]. 

## Conclusions

This case highlights the rare presentation of brain metastasis from duodenal adenocarcinoma. Brain metastasis in gastrointestinal malignancy is rare, and the cause of this biological behavior of this cancer is unclear. There are not many case reports of brain metastasis in duodenal cancer, and our case report is one of the rarer ones reported. An IHC panel was used to confirm the duodenal origin of the cancer. Our case also highlights the fact that monitoring for any new neurological findings is important in patients who continue to live longer with good systemic control of disease with chemotherapy. Current NCCN recommendations for surveillance of SBA after curative treatment are similar to colorectal cancer surveillance with history and physical, CEA and/or CA 19-9, CT of the chest, abdomen, and pelvis. Lack of data precludes a recommendation for regular surveillance brain imaging in long-surviving patients.
